# Gene expression profiles in liver of pigs with extreme high and low levels of androstenone

**DOI:** 10.1186/1746-6148-4-29

**Published:** 2008-08-06

**Authors:** Maren Moe, Sigbjørn Lien, Christian Bendixen, Jakob Hedegaard, Henrik Hornshøj, Ingunn Berget, Theo HE Meuwissen, Eli Grindflek

**Affiliations:** 1 The Norwegian Pig Breeders Association (NORSVIN), Hamar, Norway; 2 Department of Animal and Aquacultural Sciences, Norwegian University of Life Sciences, Ås, Norway; 3 Centre for Integrative Genetics (CIGENE), Norwegian University of Life Sciences, Ås, Norway; 4 Faculty of Agricultural Sciences, University of Aarhus, Tjele, Denmark; 5 Nofima Food, Oslovn 1, Ås, Norway

## Abstract

**Background:**

Boar taint is the unpleasant odour and flavour of the meat of uncastrated male pigs that is primarily caused by high levels of androstenone and skatole in adipose tissue. Androstenone is a steroid and its levels are mainly genetically determined. Studies on androstenone metabolism have, however, focused on a limited number of genes. Identification of additional genes influencing levels of androstenone may facilitate implementation of marker assisted breeding practices. In this study, microarrays were used to identify differentially expressed genes and pathways related to androstenone metabolism in the liver from boars with extreme levels of androstenone in adipose tissue.

**Results:**

Liver tissue samples from 58 boars of the two breeds Duroc and Norwegian Landrace, 29 with extreme high and 29 with extreme low levels of androstenone, were selected from more than 2500 individuals. The samples were hybridised to porcine cDNA microarrays and the 1% most significant differentially expressed genes were considered significant. Among the differentially expressed genes were metabolic phase I related genes belonging to the cytochrome P450 family and the flavin-containing monooxygenase *FMO1*. Additionally, phase II conjugation genes including UDP-glucuronosyltransferases *UGT1A5*, *UGT2A1 *and *UGT2B15*, sulfotransferase *STE*, N-acetyltransferase *NAT12 *and glutathione S-transferase were identified. Phase I and phase II metabolic reactions increase the water solubility of steroids and play a key role in their elimination. Differential expression was also found for genes encoding 17beta-hydroxysteroid dehydrogenases (*HSD17B2*, *HSD17B4*, *HSD17B11 *and *HSD17B13*) and plasma proteins alpha-1-acid glycoprotein (*AGP*) and orosomucoid (*ORM1*). 17beta-hydroxysteroid dehydrogenases and plasma proteins regulate the availability of steroids by controlling the amount of active steroids accessible to receptors and available for metabolism. Differences in the expression of *FMO1*, *NAT12*, *HSD17B2 *and *HSD17B13 *were verified by quantitative real competitive PCR.

**Conclusion:**

A number of genes and pathways related to metabolism of androstenone in liver were identified, including new candidate genes involved in phase I oxidation metabolism, phase II conjugation metabolism, and regulation of steroid availability. The study is a first step towards a deeper understanding of enzymes and regulators involved in pathways of androstenone metabolism and may ultimately lead to the discovery of markers to reduce boar taint.

## Background

Boar taint is an off-odour and off-flavour in pig carcasses that is primarily caused by high levels of 16-androstene steroids [1] and/or skatole [2] in adipose tissue. Male pigs used for meat production are normally castrated very early in life to prevent boar taint in the meat. However, castration also removes the source of natural anabolic androgens that stimulate lean growth and, as a result, uncastrated males have improved feed efficiency and greater lean yield of the carcass compared to barrows [3]. Detection of genetic factors influencing boar taint may facilitate implementation of selective breeding practices to produce pigs with little or no taint. Because of unfavourable correlations between androstenone and other sex steroids [4], a direct selection against high levels of androstenone would result in decreased production of testosterone and estrogens, with associated negative effects on performance and sexual maturation. Therefore, a comprehensive understanding of the complex genetic system controlling boar taint is required before genetic improvement can be achieved.

Androstenone (5α-androst-16-en-3-one) is produced in the testis and is transported by blood to the salivary gland where it functions as a pheromone to stimulate mating stance in gilts [5]. It is metabolised in the liver, producing α-androstenol and β-androstenol [6,7], and deficient degradation may lead to the accumulation of androstenone in adipose tissue. Skatole (3-methylindole) is produced by the metabolism of tryptophan from the gut and is also catabolised in liver. Diaz *et al*. [8] identified seven metabolites of skatole in pig liver microsomes. An increase in levels of skatole in adipose tissue occurs in boars around puberty, but not in barrows or sows, indicating that skatole metabolism is regulated by testicular steroids [9,10].

Enzymes responsible for metabolism of androstenone and skatole in liver have been identified but relatively few genes have been investigated. Several studies have shown that 3β-hydroxysteroid dehydrogenase (3β-HSD) is involved in androstenone metabolism, with low mRNA, protein and enzyme expression levels correlated to high androstenone levels in adipose tissue [6,11,12]. Cytochrome P450 2E1 (CYP2E1) is involved in metabolism of skatole [13,14] and deficient CYP2E1 induction is suggested to be a major cause of high levels of skatole in adipose tissue [10]. A relationship between metabolism of androstenone and skatole in liver has been observed [9], with evidence to suggest that androstenone blocks CYP2E1 induction by skatole [10]. Sulfotransferase enzymes including SULT2A1, SULT2B1 and SULT1A1 are associated with metabolic clearance of androstenone and skatole since they are known to conjugate steroid hormones and drugs into more water soluble compounds that facilitates excretion [12,15-20].

Although both androstenone and skatole are responsible for boar taint, androstenone seems to be the biggest problem in the Duroc and Norwegian Landrace populations [21]. Levels of androstenone are also predominantly genetically determined whereas levels of skatole are more heavily influenced by feeding and environmental factors. Levels of androstenone will be a function of both production in testes and elimination in liver. We have previously studied differentially expressed genes related to the production of androstenone in boar testis [17], and the objective of this study was to investigate global gene expression profiles related to the degradation of androstenone in pig livers. Microarray technology was used to obtain gene expression profiles from liver samples of Duroc (D) and Norwegian Landrace (NL) boars with extreme high or low levels of androstenone in adipose tissue. These samples were hybridised together with a common reference sample to cDNA microarrays representing approximately 20,000 porcine gene transcripts. A total of 29 animals were selected from each group, resulting in a total of 116 arrays. A subset of the differentially expressed genes was verified using a quantitative PCR-based method.

## Results

Porcine cDNA microarrays were used to obtain global gene expression profiles in liver tissues sampled from 58 D and 58 NL boars with extreme high and low levels of androstenone. Average levels of androstenone were 11.57 ± 3.2 ppm for D high (DH) and 0.37 ± 0.17 ppm for D low (DL) boars. Average levels of androstenone were 5.95 ± 2.04 ppm for NL high (NLH) and 0.14 ± 0.04 ppm for NL low (NLL) boars. Linear models were used to identify significantly differentially expressed genes and the top 1% (269) most significant genes were subsequently inspected as possible candidate genes affecting androstenone levels (See additional file 1: Microarray results for Duroc, additional file 2: Microarray results for Norwegian Landrace). The top 100 genes of D and NL are presented in table 1 and 2, respectively. Among the 269 affected genes, 25 were found to be in common for the two breeds and expression differences were generally more significant in D compared to NL.

**Table 1:** Top 100 genes identified in Duroc.

ID	Name	gene_id	FoldChange	adj.P.Val
103725	Cytochrome P450 2C49 (CYP2C49)	NM_214420.1	1.227	8.26e-24
104194	Cytochrome P450 2C49 (CYP2C49)	NM_214420.1	0.853	4.98e-20
102872	Glycine-N-acyltransferase (GLYAT)	NM_201648.1	1.113	1.27e-19
103265	Cytochrome P450 2C49 (CYP2C49)	NM_214420.1	1.480	4.76e-18
103234	Hepatic flavin-containing monooxygenase (FMO) (FMO1)	NM_214064.1	1.179	2.28e-17
206700	Cytochrome P450 2C49 (CYP2C49)	NM_214420.1	1.099	2.71e-16
102682	Serum amyloid P component (APCS)	NM_213887.1	1.294	2.71e-16
102284	Sterile alpha motif domain containing 8 (Samd8)	NM_026283.2	0.969	3.44e-16
102668	Hepatic flavin-containing monooxygenase (FMO) (FMO1)	NM_214064.1	1.271	8.01e-16
100748	Regulatory factor X domain containing 2 (RFXDC2)	NM_022841.3	0.594	7.47e-14
206864	Similar to Alcohol dehydrogenase 6 (LOC523510)	XM_601810.2	-0.925	3.49e-13
102390	Cytochrome P450 2C49 (CYP2C49)	NM_214420.1	1.152	5.05e-13
102098	Carnitine O-octanoyltransferase (CROT)	NM_021151.2	0.886	5.30e-13
102991	Cytochrome P450. family 2. subfamily E. polypeptide 1 (CYP2E1)	NM_214421.1	-1.040	2.17e-12
216026	216026		-0.728	3.72e-12
102413	Small nuclear RNA activating complex. polypeptide 1. 43 kDa (SNAPC1)	NM_003082.2	0.994	4.84e-12
103432	103432		0.527	2.07e-11
210250	Metallothionein (MT1A)	NM_001001266.1	-0.965	3.09e-11
103062	FKBP1A-like (LOC654323)	NM_001038000.1	-1.078	3.09e-11
103279	Orosomucoid 1 (ORM1)	NM_000607.1	-0.947	3.99e-11
103021	Hydroxysteroid (17-beta) dehydrogenase 13 (HSD17B13)	NM_178135.2	-0.869	6.10e-11
201488	201488		0.462	1.08e-10
100575	17beta-estradiol dehydrogenase (HSD17B4)	NM_214306.1	0.452	1.24e-10
100723	Aspartate aminotransferase (GOT2)	NM_213928.1	0.493	3.71e-10
103194	Alpha-1 acid glycoprotein (AGP)	XM_585370.2	-0.932	4.52e-10
202082	Family with sequence similarity 10. member A4 (FAM10A4)	NR_002183.1	0.424	6.45e-10
100724	Phytanoyl-CoA hydroxylase (PHYH)	NM_006214.3	0.518	8.69e-10
210077	Cytochrome P450 2C49 (CYP2C49)	NM_214420.1	0.872	9.95e-10
101277	Estrogen sulfotransferase (STE)	NM_213992.1	0.557	1.45e-09
216031	Insulin-like-growth factor 2 (IGF2)	NM_213883.1	-0.687	1.88e-09
210204	Similar to HLA class I histocompatibility antigen, A-11 alpha chain precursor (LOC642049)	XM_936199.1	-0.591	1.88e-09
103031	Type I iodothyronine deiodinase (DIO1)	NM_001001627.1	0.499	2.85e-09
210252	Sarcosine dehydrogenase (SARDH)	NM_007101.2	-0.273	3.55e-09
102976	Similar to UDP-glucuronosyltransferase 2B15 precursor (UDPGT) (HLUG4) (LOC653180)	XM_931558.1	0.623	4.69e-09
201274	Sorbitol dehydrogenase (SORD). mRNA	NM_003104.3	0.523	5.70e-09
103801	Similar to Sorbitol dehydrogenase (L-iditol 2-dehydrogenase) (LOC650043)	XM_939131.1	0.452	1.83e-08
204461	Glycine-N-acyltransferase (GLYAT). nuclear gene encoding mitochondrial protein	NM_005838.2	0.370	2.47e-08
102538	102538		0.746	2.65e-08
204863	Hydroxysteroid (17-beta) dehydrogenase 13 (HSD17B13)	NM_178135.2	-0.630	3.89e-08
101843	Abhydrolase domain containing 3 (ABHD3)	NM_138340.3	0.370	9.87e-08
215880	Transcription elongation factor B (SIII). polypeptide 3 (110 kDa. elongin A) (TCEB3)	NM_003198.1	-0.612	9.87e-08
200322	Taxilin beta (TXLNB)	NM_153235.2	0.385	9.87e-08
210206	Metallothionein (MT1A)	NM_001001266.1	-0.536	1.25e-07
201503	Carboxylesterase (CES3)	NM_214246.1	0.472	1.44e-07
202070	202070		0.485	1.57e-07
102106	102106		0.335	1.86e-07
206300	Protein tyrosine phosphatase type IVA. member 1 (PTP4A1)	NM_003463.3	0.337	2.01e-07
103047	Similar to mouse 2310016A09Rik gene (LOC134147)	NM_138809.3	0.456	2.01e-07
204653	FKBP1A-like (LOC654323)	NM_001038000.1	0.256	2.06e-07
103327	UDP glucuronosyltransferase 1 family, polypeptide A5 (UGT1A5)	NM_019078.1	0.264	2.42e-07
102469	Chromosome 1 open reading frame 128 (C1orf128)	NM_020362.2	0.190	2.42e-07
201919	FKBP1A-like (LOC654323)	NM_001038000.1	0.303	2.53e-07
103349	Kininogen 1 (KNG1)	NM_000893.2	0.428	3.03e-07
103940	UDP glucuronosyltransferase 1 family, polypeptide A5 (UGT1A5)	NM_019078.1	0.191	4.03e-07
204455	Ornithine carbamoyltransferase (OTC)	NM_000531.3	0.357	4.61e-07
212598	L1 cell adhesion molecule (L1CAM)	NM_024003.1	-0.167	4.72e-07
207639	207639		0.383	4.83e-07
200534	200534		0.388	4.89e-07
102183	Protein tyrosine phosphatase type IVA. member 1 (PTP4A1)	NM_003463.3	0.360	5.72e-07
206424	Similar to transcription elongation factor B polypeptide 3 binding protein 1 isoform 1 (LOC533427)	XM_612827.2	0.265	6.41e-07
103306	Tryptophan 2.3-dioxygenase (TDO2)	NM_005651.1	0.443	6.41e-07
104125	Sorbitol dehydrogenase (SORD)	NM_003104.3	0.428	7.73e-07
102709	102709		0.444	7.76e-07
100472	S-adenosylhomocysteine hydrolase (AHCY)	NM_001011727.1	0.321	8.64e-07
206129	206129		-0.484	8.86e-07
102624	Ornithine carbamoyltransferase (OTC)	NM_000531.3	0.393	8.86e-07
213735	Metallothionein (MT1A)	NM_001001266.1	0.422	1.01e-06
102836	Carboxylesterase (CES3)	NM_214246.1	0.563	1.10e-06
201162	FKBP1A-like (LOC654323)	NM_001038000.1	0.268	1.42e-06
201961	201961		0.293	1.42e-06
216074	Metallothionein (MT1A)	NM_001001266.1	-0.772	1.64e-06
207278	207278		0.282	1.67e-06
101716	101716		0.328	1.75e-06
204421	Meiosis-specific nuclear structural 1 (MNS1)	NM_018365.1	0.364	1.99e-06
103156	103156		0.333	2.05e-06
103376	Hydroxysteroid (17-beta) dehydrogenase 13 (HSD17B13)	NM_178135.2	-0.497	2.38e-06
100064	Similar to UDP-galactoseN-acetylgalactosamine-alpha-R beta 1,3-galactosyltransferase (LOC645551)	XM_928571.1	0.159	2.43e-06
204950	Abhydrolase domain containing 3 (ABHD3)	NM_138340.3	0.236	2.43e-06
204048	Suppression of tumorigenicity 13 (colon carcinoma) (Hsp70 interacting protein) (ST13)	NM_003932.3	0.379	2.74e-06
204867	204867		0.308	2.95e-06
207963	207963		0.254	3.08e-06
200952	200952		0.236	3.08e-06
103294	Similar to Mob4B protein (MGC124888)	NM_001033891.1	0.369	3.79e-06
208427	RAR-related orphan receptor A (RORA)	NM_134262.1	0.246	4.25e-06
208058	FKBP1A-like (LOC654323)	NM_001038000.1	0.247	4.48e-06
205360	FLJ20105 protein (FLJ20105)	NM_017669.2	0.278	4.73e-06
207838	3-hydroxymethyl-3-methylglutaryl-Coenzyme A lyase (hydroxymethylglutaricaciduria) (HMGCL)	NM_000191.2	0.156	4.78e-06
101413	Replication protein A3. 14 kDa (RPA3)	NM_002947.3	0.253	5.25e-06
103180	T-complex 1 (TCP1)	NM_001008897.1	0.310	5.69e-06
202298	202298		0.262	5.73e-06
100384	Alpha-methylacyl-CoA racemase (AMACR)	NM_014324.4	0.394	6.68e-06
103434	Acyl-Coenzyme A oxidase 1, palmitoyl (ACOX1)	NM_004035.5	0.368	7.11e-06
202477	Hypothetical LOC505518 (LOC505518)	XM_581816.2	0.240	7.42e-06
102858	102858		0.587	7.52e-06
103058	103058		0.381	7.67e-06
211612	Gamma-aminobutyric acid (GABA) A receptor. alpha 3 (GABRA3)	NM_000808.2	-0.135	8.97e-06
201347	HRAS-like suppressor 3 (HRASLS3)	NM_007069.1	0.352	8.97e-06
102840	Sorbitol dehydrogenase (SORD)	NM_003104.3	0.270	8.97e-06
201105	COP9 constitutive photomorphogenic homolog subunit 2 (Arabidopsis) (COPS2)	NM_004236.1	0.242	9.06e-06
201848	Kinesin family member C1 (KIFC1)	NM_002263.2	0.274	9.06e-06

Gene expression profiles were identified in Limma using empirical Bayes moderated t-statistics. Fold change and statistical significance is shown (FDR-adjusted p-values). The clone names refer to hits to pig, human, mouse or bovine sequences. Some genes are represented by several different clones on the array and may therefore show up more than once in the table, while others have no hits to the abovementioned species.

**Table 2:** Top 100 genes identified in Norwegian Landrace.

ID	Name	gene_id	FoldChange	adj.P.Val
206864	Similar to Alcohol dehydrogenase 6 (LOC523510)	XM_601810.2	-0.730	1.07e-13
204853	204853		-0.441	2.39e-13
209551	Isochorismatase domain containing 1 (ISOC1)	NM_016048.1	-0.403	3.28e-13
103021	Hydroxysteroid (17-beta) dehydrogenase 13 (HSD17B13)	NM_178135.2	-0.900	3.28e-13
204863	Hydroxysteroid (17-beta) dehydrogenase 13 (HSD17B13)	NM_178135.2	-0.730	3.78e-13
209525	Zinc finger protein 471 (ZNF471)	NM_020813.1	-0.335	1.69e-12
102789	102789		-0.364	1.69e-12
204725	204725		-0.367	1.69e-12
103376	Hydroxysteroid (17-beta) dehydrogenase 13 (HSD17B13)	NM_178135.2	-0.589	2.41e-12
205190	Similar to heterogeneous nuclear ribonucleoprotein C isoform b (LOC654074)	XM_945342.1	0.249	7.01e-12
204302	Aldehyde oxidase 1 (AOX1)	NM_001159.3	-0.481	9.60e-12
204466	204466		-0.345	1.20e-11
203752	203752		-0.274	1.52e-11
206901	Non-histone protein HMG1 (LOC445521)	NM_001004034.1	-0.286	5.34e-11
218781	KIAA1344 (KIAA1344)	NM_020784.1	0.280	5.34e-11
216031	Insulin-like-growth factor 2 (IGF2)	NM_213883.1	-0.847	5.34e-11
213651	213651		-0.437	9.01e-11
215880	Transcription elongation factor B (SIII). polypeptide 3 (110 kDa. elongin A) (TCEB3)	NM_003198.1	-0.797	9.45e-11
204619	Cytochrome b-5 (CYB5)	NM_001001770.1	-0.458	9.45e-11
204520	204520		-0.290	1.45e-10
103177	Cytochrome b-5 (CYB5)	NM_001001770.1	-0.489	2.06e-10
102774	Non-histone protein HMG1 (LOC445521)	NM_001004034.1	-0.302	3.48e-10
204081	204081		-0.251	3.48e-10
100528	ATP synthase. H+ transporting. mitochondrial F0 complex. subunit F6 (ATP5J)	NM_001003703.1	0.250	3.55e-10
211561	KIAA0427 (KIAA0427)	NM_014772.1	0.263	4.63e-10
209674	209674		0.269	7.12e-10
208386	Dual specificity phosphatase 12 (DUSP12)	NM_007240.1	-0.318	7.12e-10
207228	Small nuclear ribonucleoprotein polypeptide E (SNRPE)	NM_003094.2	0.252	7.37e-10
219297	Purine-rich element binding protein A (PURA)	NM_005859.3	0.499	8.70e-10
218205	SMC1 structural maintenance of chromosomes 1-like 1 (yeast) (SMC1L1)	NM_006306.2	0.300	1.11e-09
103194	Alpha-1 acid glycoprotein (AGP)	XM_585370.2	-1.071	1.16e-09
219538	219538		0.352	1.67e-09
201622	Hypothetical protein LOC285016 (LOC285016)	NM_001002919.1	-0.417	1.99e-09
217117	Amyloid beta (A4) precursor protein-binding. family B. member 3 (APBB3)	NM_006051.2	0.294	2.38e-09
102677	4-aminobutyrate aminotransferase (ABAT)	NM_000663.2	0.288	2.38e-09
216026	216026		-0.840	2.51e-09
216643	Leupaxin (Lpxn)	NM_134152.1	0.271	2.93e-09
205721	205721		-0.256	3.02e-09
202919	202919		0.272	3.02e-09
220257	Similar to 60S ribosomal protein L39 (LOC651724)	XM_940942.1	0.273	3.10e-09
103136	Mitochondrial translational initiation factor 2 (MTIF2)	NM_001005369.1	-0.250	3.35e-09
201942	201942		-0.286	4.06e-09
216638	216638		0.304	4.06e-09
204605	Similar to Maltase-glucoamylase, intestinal (LOC642103)	XM_936233.1	-0.234	4.12e-09
208567	208567		-0.307	4.71e-09
103361	Phenylalanine hydroxylase (PAH)	NM_000277.1	-0.627	5.15e-09
202659	FKBP1A-like (LOC654323)	NM_001038000.1	-0.312	5.49e-09
207982	La ribonucleoprotein domain family. member 4 (LARP4)	NM_052879.3	-0.247	5.63e-09
204513	Deoxyribonuclease II (DNASE2)	NM_214196.1	-0.255	5.73e-09
206370	Syndecan binding protein (syntenin) (SDCBP)	NM_001007068.1	-0.208	6.33e-09
214304	Heat shock transcription factor 1 (HSF1)	NM_005526.1	0.331	7.28e-09
103062	FKBP1A-like (LOC654323)	NM_001038000.1	-1.046	7.37e-09
200969	Family with sequence similarity 70. member A (FAM70A)	NM_017938.2	0.424	7.37e-09
206063	Chromosome 14 open reading frame 43 (C14orf43)	NM_194278.2	-0.230	8.05e-09
206129	206129		-0.447	1.10e-08
206577	Leukemia inhibitory factor receptor (LIFR)	NM_002310.3	-0.265	1.39e-08
103378	Similar to Putative steroid dehydrogenase KIK-I (LOC508455)	XM_585231.2	-0.314	1.49e-08
204838	FKBP1A-like (LOC654323)	NM_001038000.1	-0.309	1.49e-08
104413	Pyruvate carboxylase (PC)	NM_214349.1	0.246	1.49e-08
219405	Coiled-coil domain containing 42 (CCDC42)	NM_144681.1	0.447	1.49e-08
209512	209512		-0.310	1.72e-09
221025	221025		0.446	1.74e-08
200362	Chromosome 3 open reading frame 43, transcript variant 1 (C3orf43)	XM_173087.5	-0.199	1.88e-08
206386	206386		-0.262	2.65e-08
211845	Alanyl-tRNA synthetase domain containing 1 (AARSD1)	NM_025267.2	0.250	3.31e-08
208761	Ring finger protein 148 (RNF148)	NM_198085.1	-0.189	3.31e-08
209449	Similar to Zinc finger CCCH-type domain containing protein 11A transcript variant 3 (LOC441155)	XM_930970.1	-0.237	3.31e-08
205142	Bone morphogenetic protein receptor, type IA (BMPR1A)	NM_004329.2	-0.340	3.31e-08
203722	203722		-0.217	3.32e-08
103279	Orosomucoid 1 (ORM1)	NM_000607.1	-0.999	3.32e-08
101898	Bifunctional apoptosis regulator (BFAR)	NM_016561.1	-0.189	3.32e-08
104972	Cyclin-dependent kinase inhibitor 3 (CDKN3)	NM_214320.1	0.364	3.45e-08
202997	202997		-0.270	3.45e-08
102991	Cytochrome P450. family 2. subfamily E. polypeptide 1 (CYP2E1)	NM_214421.1	-1.079	3.68e-08
208187	Early growth response 3 (EGR3)	NM_004430.2	0.378	3.77e-08
205347	Histone H3.3A (H3F3A)	NM_213930.1	-0.237	3.77e-08
203762	Zinc finger protein 7 (KOX 4. clone HF.16) (ZNF7)	NM_003416.1	-0.234	4.37e-08
102955	L-kynurenine 3-monooxygenase Fpk (KMO)	NM_214076.1	-0.294	4.81e-08
212532	TBC1 domain family. member 10B (TBC1D10B)	NM_015527.2	0.231	4.99e-08
206466	Similar to glycosyltransferase-like domain containing 1 isoform a (LOC534677)	XM_871633.1	-0.299	5.15e-08
104900	Cullin 4A (CUL4A). transcript variant 1	NM_001008895.1	0.349	5.59e-08
208650	FKBP1A-like (LOC654323)	NM_001038000.1	-0.214	5.83e-08
205233	5'-3' exoribonuclease 2 (XRN2)	NM_012255.3	0.217	5.99e-08
102672	Cytochrome b-5 (CYB5)	NM_001001770.1	-0.528	6.21e-08
101034	Programmed cell death 6 interacting protein (PDCD6IP)	NM_013374.3	-0.247	6.21e-08
206253	Cyclin-dependent kinase 6 (CDK6)	NM_001259.5	-0.260	6.21e-08
209516	209516		-0.208	6.21e-08
100909	Myeloid-associated differentiation marker (MYADM)	NM_001020819.1	-0.239	7.42e-08
205225	Family with sequence similarity 80, member B (FAM80B)	NM_020734.1	0.247	7.42e-08
200683	200683		-0.302	7.64e-08
208724	Dickkopf-like 1 (soggy) (DKKL1)	NM_014419.3	0.275	7.64e-08
218253	Mesoderm induction early response 1, family member 2 (MIER2)	NM_017550.1	0.265	7.97e-08
209231	Heparin binding protein (HBP15/L22)	NM_213987.1	-0.343	8.00e-08
104000	Ribosomal protein L39 (RPL39)	NM_001000.2	0.264	8.37e-08
205725	205725		-0.208	8.73e-08
102726	Complement factor I (CFI)	NM_000204.1	-0.380	8.77e-08
100609	Proline rich 13 (PRR13)	NM_001005355.1	0.267	9.03e-08
104957	Succinyl-CoA:alpha-ketoacid coenzyme A transferase (OXCT)	NM_213938.1	0.392	9.03e-08
216727	216727		-0.287	9.12e-08
200320	Ubiquitin specific peptidase 32 (USP32)	XM_939750.1	0.439	9.28e-08

Gene expression profiles were identified in Limma using empirical Bayes moderated t-statistics. Fold change and statistical significance is shown (FDR-adjusted p-values). The clone names refer to hits to pig, human, mouse or bovine sequences. Some genes are represented by several different clones on the array and may therefore show up more than once in the table, while others have no hits to the abovementioned species.

**Figure 1:**
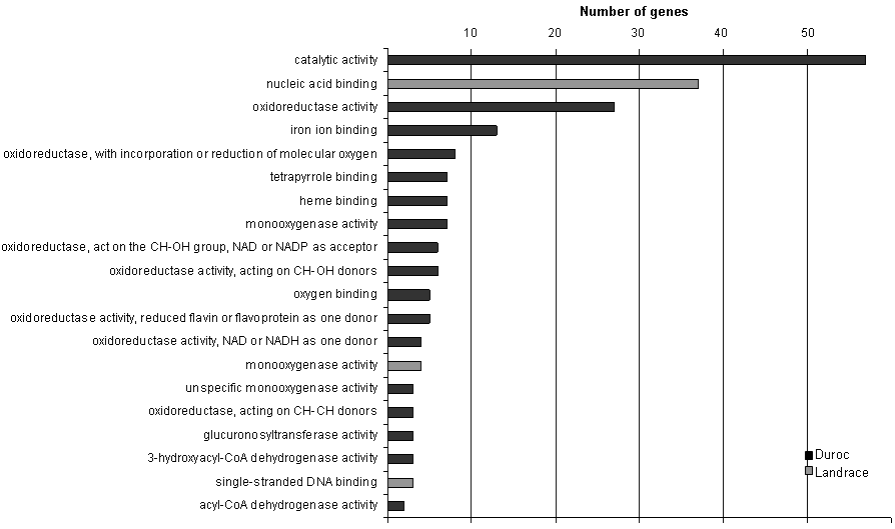


**Figure 2:**
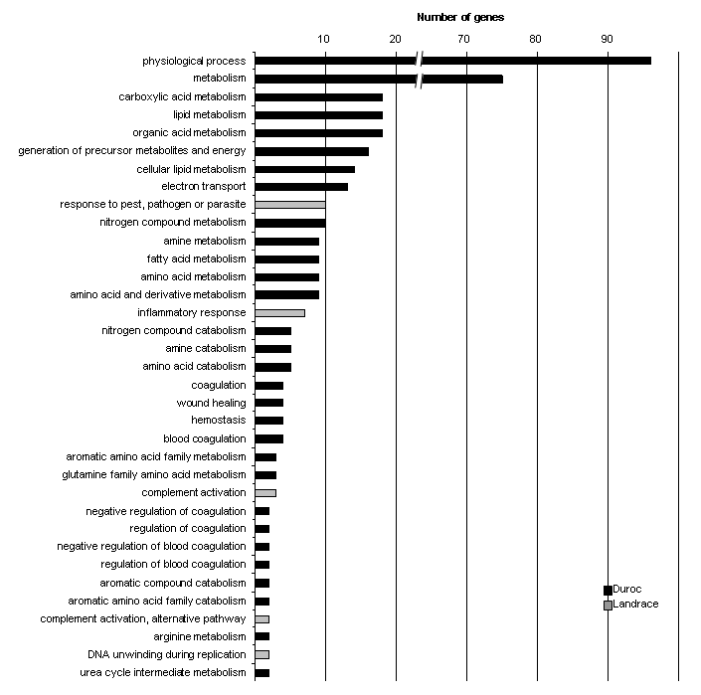


Testing for overrepresentation of gene ontology (GO) terms among the 269 affected genes was performed relative to the global representation of GO terms on the microarray. A majority of the significant genes were related to oxidoreductase activities, glucuronosyltransferase activity and the binding of iron ion, haem, DNA and oxygen (Figure 1). Additionally, cellular metabolic process pathways such as amino acid metabolism and catabolism, electron transport and inflammatory response were overrepresented (Figure 2). Furthermore, the genes were classified according to their cellular component ontology (See additional file 3: Gene ontology (GO) results for the cellular component ontology in Duroc, additional file 4: Gene ontology (GO) results for the cellular component ontology in Norwegian Landrace). For D, cytoplasm, microsomes and endoplasmatic reticulum were among the important cellular components, none of the cellular components reached the level of significance, i.e. had more than 10 significant genes related to the CC terms.

**Figure 3:**
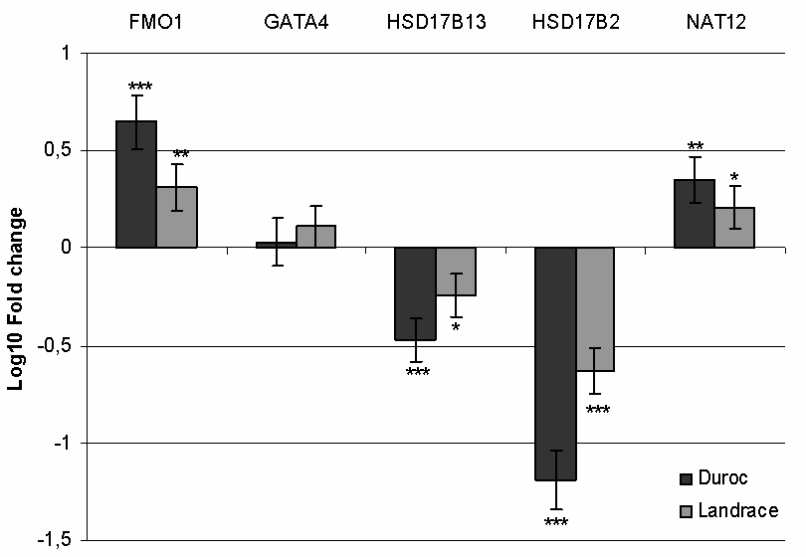


To validate the microarray results, a real competitive PCR (rcPCR) approach was applied to five selected genes: Flavin-containing monooxygenase 1 (*FMO1*), transcription factor GATA-4 (*GATA4*), 17β-hydroxysteroid dehydrogenase 13 (*HSD17B13*), 17β-hydroxysteroid dehydrogenase 2 (*HSD17B2*) and N-acetyltransferase 12 (*NAT12*). Their expression levels were normalised to the housekeeping gene transferrin receptor (*TFRC*). Differential expression of *FMO1*, *HSD17B13*, *HSD17B2 *and *NAT12 *was confirmed in both D and NL breeds (p < 0.05) while altered expression of *GATA4 *was not found in either of the two breeds (Figure 3).

## Discussion

Elevated levels of androstenone in adipose tissue can result from both increased biosynthesis in the testes and deficient metabolism in liver. We have previously conducted a microarray experiment investigating gene expression profiles in the testes of boars with extreme levels of androstenone, and in this study we have examined gene expression profiles in the liver of some representatives from that study along with new individuals. Liver metabolism can be divided into phase I and phase II reactions [22]. Phase I metabolism involves oxidation and hydroxylation reactions that make the substrate more water soluble. Phase II conjugation reactions further increase hydrophilicity by adding polar groups. As a result of these metabolic reactions, compounds including endogenous steroids, fatty acids and drugs are inactivated and eliminated. We have identified a number of differentially expressed genes that function in pathways affecting both phase I and phase II reactions involved in metabolism of androstenone in the liver.

### Phase I metabolism

The most significantly differentially expressed gene involved in phase I oxidation reactions was cytochrome P450 2C49 (*CYP2C49*). This gene is a member of the CYP2C family [23] and genes of this family encode monooxygenases that catalyse several reactions involved in the metabolism of drugs and endogenous compounds. Different CYP2C isoforms show some cross reactivity towards substrates which makes it difficult to differentiate CYP2C activities, and substrate specificity for CYP2C49 is not known [24]. In our study, *CYP2C49 *transcripts were shown to have a significant up-regulation in boars with high levels of androstenone in both D and NL lines (DH and NLH) but differences were clearly most prominent in the D breed.

Another member of the cytochrome P450 superfamily, that was differentially expressed in our study, is cytochrome P450 2E1 (*CYP2E1*). In contrast to expression of the *CYP2C49 *gene, *CYP2E1 *showed a down-regulation in DH and NLH boars. *CYP2E1 *encodes an enzyme that metabolises many endogenous and exogenous substrates such as alcohols, ketones and drugs [24]. Androstenone has been shown to block skatole induced expression of CYP2E1 in pig hepatocytes and to inhibit activity of CYP2E1 in porcine liver microsomes [10,25]. Liver metabolism of skatole also involves this gene and enzyme [13,14,26-28], and the observed pubertal increase in skatole levels has been attributed to the inhibition of CYP2E1 by the sex steroids androstenone and 17β-estradiol [25]. This is the first study, however, to show gene expression differences for *CYP2E1 *in relation to androstenone.

Cytochrome P450 member 2A19 (*CYP2A19*), which is the pig ortholog of human *CYP2A6*, catalyses 7-hydroxylation of coumarin in pigs [26], in addition to being involved in skatole metabolism [29]. In this study, *CYP2A19 *was significantly down-regulated in DH boars. Gene expression differences in animals with high and low androstenone levels have not previously been reported for porcine *CYP2A19*, however its protein expression has been shown to be inhibited by androstenone [30]. Diaz and Squires [26] found that both CYP2A6 protein content and its enzymatic activity were negatively correlated with skatole levels in adipose tissue. In contrast, results presented by Terner *et al*. [28] indicate that the CYP2A6 enzyme is not important for metabolism of skatole in primary cultured porcine hepatocytes.

Two other members of the cytochrome P450 family, *CYP27A1 *and *CYP2C33*, were also up-regulated in DH. CYP27A1 is a multifunctional enzyme that oxygenates cholesterol, bile acids and vitamin D. It has not previously been identified as a candidate gene associated with androstenone, but it is regulated by other sex steroids in human cells [31]. *CYP2C33 *belongs to the same sub-family as *CYP2C49*, but its function has yet to be described in the literature. Like the *CYP2C49 *gene, *CYP2C33 *was found to be up-regulated in DH and NLH boars. The differential expression of *CYP2A19, CYP27A1 *and *CYP2C33 *in D and not NL boars might suggest that more genes are involved in phase I reactions in D. This is supported by a high number of oxidoreductase pathways significant in D (figure 1). Monooxygenase reactions are, however, clearly important in both breeds according to our gene ontology results (figure 1). Down-regulation of *CYP2E1 *and *CYP2A19 *might suggest a different role for these genes compared to the up-regulated *CYP2C18*, *CYP2C33 *and *CYP27A1*.

Our study implicates another class of genes involved in phase I oxidation reactions, namely the flavin-containing monooxygenases (FMOs). The FMO family of enzymes converts lipophilic compounds into more polar metabolites and decreases activity of the compounds, a similar activity to that of the cytochrome P450s [32]. Microarray expression results show that flavin-containing monooxygenase 1 (*FMO1*) was significantly up-regulated in DH in the microarray results. The expression pattern in DH boars was confirmed by rcPCR and the rcPCR analysis also revealed that *FMO1 *was up-regulated in NLH boars. As shown in figure 2, the fold change in NLH was only half of DH, which might explain why this gene was not found to be significantly differentially expressed in the microarray results. Regulation of FMO involves sex steroids. In male mice, castration was reported to increase FMO1 expression [33], while in rats the opposite effect has been shown, with positive regulation of FMO by testosterone and negative by estradiol [34]. The monooxygenase activity of FMO1 in addition to its regulation by steroid hormones makes it an interesting candidate gene for boar taint.

### Phase II metabolism

Phase I oxidation reactions of androstenone by enzymes including cytochrome P450s and FMOs are often followed by phase II conjugation reactions that are catalysed by glucuronosyltransferases, sulfotransferases, acetyltransferases and glutathione S-transferases [22]. Through the addition of polar moieties, these enzymes increase substrate solubility. Conjugation reactions are an important means of excreting steroid hormones and other compounds, these reactions have also been proposed to keep inactive steroids easily available in cells. The hydroxysteroid sulfotransferases SULT2A1 and SULT2B1 have been associated with androstenone levels in previous studies [12,15-17]. These genes were not found to be significantly differentially expressed in our study, however the estrogen sulfotransferase *STE *(*SULT2E1*) was found to be up-regulated in DH boars and down-regulated in NLH boars. A previous microarray study performed by our group showed an up-regulation of *CYP19 *gene expression in the testis of D and NL boars with high androstenone levels [17]. These genes encode enzymes biosynthesising estrogens and might explain why an estrogen conjugation enzyme is differentially expressed in livers of the same pigs. Furthermore, boars secrete large quantities of estrogens from the testis [35], and both fat and plasma estrogen levels have previously been shown to be highly correlated with levels of androstenone in adipose tissue [36,37].

Phase II conjugation by glucuronidation is another major pathway of liver elimination of endogenous and exogenous compounds, and in D this molecular function was significantly overrepresented. It is catalysed by uridine diphospho-glucuronosyltransferases (UGTs) which transfer glucuronic acid to substrates to increase solubility [38]. The UGTs have been divided into two subfamilies, the UGT1s and the UGT2s, and in this study the family member *UGT1A5 *was found to be up-regulated in DH boars. Furthermore, a transcript similar to *UGT2B15 *was up-regulated in D and a transcript similar to *UGT2A1 *was down-regulated in NLH. UGT2B15 has been shown to conjugate several androgens in humans [38], whereas UGT1A5 has been found to be catalytically active against some exogenous compounds [39]. UGT2A1 contributes to glucuronidation of steroids and phenolic compounds in olfactory tissue and has been found to eliminate odourants [40]. The UGT family of conjugation enzymes have previously been associated with the androstenone metabolites α-androstenol and β-androstenol [41,42].

N-acetyltransferases (NATs) are another family of conjugation enzymes and act by transferring the acetyl group of acetyl coenzyme A to aromatic amines to increase their water solubility [22]. NAT type 12 (*NAT12*) was found to be up-regulated in NLH based on the microarray results and this was verified by rcPCR. The rcPCR results also revealed an up-regulation in DH boars. Androgens have been shown to increase expression of NAT type 1 in human cancer cells [43], but the enzyme family has not previously been associated with levels of androstenone. Tryptophan, the precursor of skatole, contains an aromatic, suggesting a role for this family of conjugation enzymes in the control of skatole levels, possibly through regulation by steroid hormones.

Glutathione S-transferases (GSTs) are functionally diverse enzymes mostly known to catalyse conjugation reactions of endogenous substances, haem, fatty acids, xenobiotics and products of oxidative processes [44]. They have also been implicated in the intracellular transport of steroids to their site of action [44]. In this study, a glutathione S-transferase gene was found to be down-regulated in NLH. The gene is described in the database as *LOC396850*. We have previously described up-regulation of two GST genes, *GSTO1 *and *MGST1*, in association with testicular androstenone levels [17], and here we additionally propose a role for glucuronidation in liver metabolism. Expression of phase II metabolic genes in D and NL pigs suggest breed specific mechanisms. We found differential expression of a GST variant (*LOC396850*) in NL but not D boars, up-regulation of *STE *in DH but down-regulation in NLH, and up-regulation of two *UGT *genes in DH but down-regulation of another family member in NLH. Breed specific phase II mechanisms for these breeds are in agreement with our previous finding for the protein *SULT2B1 *[12].

### Regulation of steroid availability

17β-hydroxysteroid dehydrogenases (17β-HSDs) regulate the availability of androgens and estrogens in tissues by catalysing interconvertion of active and inactive forms of steroids [45]. They do so by regulating the occupancy of steroid nuclear hormone receptors like androgen receptor, estrogen receptor and progesterone receptor [46]. Gene expression of *17*β-HSD in porcine liver has been shown to be negatively associated with levels of androstenone in adipose tissue [37]. In a previous study, we found that the *HSD17B4 *gene was significantly up-regulated in testicle samples from DH and NLH [17], and in this study *HSD17B4 *was up-regulated in the liver of DH boars. Additionally, we found that *HSD17B2 *was down-regulated in DH and that the isoform *HSD17B13 *was down-regulated in both DH and NLH. Differential expression of both *HSD17B2 *and *HSD17B13 *was verified by rcPCR, which also showed significant down-regulation of *HSD17B2 *in NLH boars. The HSD17B2 enzyme catalyses the interconversion of testosterone and androstenedione, as well as estradiol and estrone [47]. The function of isoform *HSD17B13*, also known as short-chain dehydrogenase/reductase 9, has yet to be described but its expression has been characterised in human liver [48]. The short-chain dehydrogenase/reductase 8 gene (*DHRS8*), also known as HSD17B11, was found to be down-regulated in NLH boars. HSD17B11 is involved in androgen metabolism [49] and we previously found this gene to be down-regulated in the testis of DH boars [17]. The HSDs belong to two superfamilies: the short-chain dehydrogenase/reductases (SDRs) and the aldo-keto reductases (AKRs) [46]. The four HSDs described above all belong to the SDR superfamily but a gene from the AKRs was also found to be differentially expressed. The aldo-keto reductase family member 1D1 (*AKR1D1*) was down-regulated in NLH animals. AKR1D1 is a liver specific enzyme that regulates the hormone levels of several steroids [46]. The enzyme has a 5β-reductase activity and is necessary for the catabolism of aldosterone, cortisol and androgens [50]. Aldosterone, cortisol and androgens are three of four metabolic compounds formed from pregnenolone, with the fourth compound being androstenone [5]. A role for AKR1D1 in androstenone catabolism might therefore be possible. We previously reported up-regulation of the family member *AKR1C4 *in the testis of DH and NLH boars [17], but *AKR1D1 *has not formerly been associated with porcine androstenone levels.

Another class of enzymes that regulate the availability of steroids is the plasma proteins. Binding of plasma protein is a reversible reaction that has no physiological effect, but it controls the amount of free drugs and hormones available to tissues by protecting them from metabolism [51]. Alpha-1-acid glycoprotein (AGP) is an acute phase serum protein synthesised in the liver and secreted to plasma where it binds and carries drugs and steroid hormones [51]. The protein has been shown to interact with CYP3A and CYP2C19 in humans and to inhibit cytochrome P450 activity [52,53]. It is suggested that serum protein interaction is an important factor for cytochrome P450 mediated metabolism, and that the interaction is isoform specific [53]. AGP has also been studied in pigs as a binding factor of pheromones, but has not been found to bind progesterone in pig nasal mucosa [54]. AGP has two variants in humans: orosomucoid 1 and 2 (ORM1 and ORM2) [51]. In this study we found that porcine transcript homologous to bovine *AGP *and human *ORM1 *were significantly down-regulated in DH and NLH boars. Significant expression of these genes might explain the inflammatory response ontology term in figure 2 as these genes have mostly been described in relation to inflammatory response. AGP has not been investigated as a binding protein and transporter for androstenone, but different levels of androstenone could be related to differences in its availability through plasma binding by AGP.

### Regulatory factors

One of the most significant molecular function terms in NL was nucleic acid binding. This term includes transcription factor binding and several transcription factors were found to be differentially expressed in this study. The GATA factor 4 (*GATA4*) was found to be down-regulated based on the microarray data, but we were not able to confirm this result with rcPCR. Other transcription factors identified in this study should be further investigated, including RAR-related orphan receptor A (*RORA*), transcription elongation factor B3 (*TCEB3*), nuclear factor I/X (*NFIX*), transcription factor 8 (*TCF8*) and heat shock transcription factor 1 (*HSF1*). We have previously identified iron ion binding, ferric ion binding and electron transport as being associated with levels of androstenone [17], possibly through interaction with the haem-containing cytochrome P450s. In this study we also found iron ion binding and electron transport, in addition to haem binding, to be important pathways, supporting our previous findings. Cytochrome b5 (CYB5) is involved in electron transfer to cytochrome P450s and has been proposed as a candidate gene for androstenone through its interaction with cytochrome P450 c17 (CYP17) [55]. In this study we found *CYB5 *to be down-regulated in NLH boars, however we previously identified *CYB5 *as being up-regulated in the testis of both DH and NLH [17]. Both *CYP2E1 *and *CYP2A6 *have been found to be activated by *CYB5 *in humans [56], supporting the down-regulation of *CYB5 *together with *CYP2E1 *and *CYP2A6 *in this study. Up-regulation in testis and down-regulation in liver might also support a key regulatory role of *CYB5 *in both tissues.

### Breed differences

Breed differences in boar taint candidate genes have previously been shown between D and NL [12,17]. Consistent with these findings, we found breed differences in the expression profiles of genes involved in both phase I metabolism and phase II metabolism. However, rcPCR results might suggest that genes appearing in one breed can be differentially expressed also in the other breed. Additional and more comprehensive rcPCR studies are planned to clarify this. In general, the D breed showed higher levels of significance compared to the NL breed, potentially due to the larger contrasts between high and low androstenone groups in this breed. A number of new candidate genes have been identified in this study, however, we do not know their effect on performance and sexual maturation in pig. Additional studies are needed to investigate potential roles of the genes identified for phenotypes related to fertility.

### Relationship with skatole

Pigs in this study were selected based on extreme androstenone values. However, levels of androstenone and skatole have previously been found to be highly correlated, showing genetic correlations of 0.62 for D and 0.36 for NL [21]. Some of the genes found differentially expressed in this study have previously been found to be associated with tryptophan or skatole and this could be explained by high correlations with androstenone. This might also be the reason for the significance of GO terms such as aromatic amino acid metabolism and catabolism, since the skatole precursor tryptophan belongs to this family of amino acids. The tryptophan 2, 3-dioxygenase gene (*TDO2*), which oxidises tryptophan, was differentially expressed in DH. A gene of the FMO family, kynurenine 3-monooxygenase (*KMO*), was found to be down-regulated in NLH. KMO is involved in tryptophan degradation and might therefore be interesting in regards to skatole [57]. The aldehyde oxidase (*AOX1*) gene was significantly down-regulated in high androstenone animals of both breeds and this gene has not previously been associated with androstenone levels. It has, however, been shown to play an important role in skatole metabolism in several species including pigs [58-60].

## Conclusion

In this study we compared global gene expression profiles from the livers of boars with extreme high and low levels of androstenone from two breeds, Duroc and Norwegian Landrace. Breed differences are evident for molecular functions and biological processes involved in metabolism of androstenone, however many of the same genes are differentially expressed in the two breeds as well. Genes encoding different oxidising enzymes including the cytochrome P450 family (*CYP2E1*, *CYP2A19*, *CYP2C49*, *CYP27A1 *and *CYP2C33*) and the flavin-containing monooxygenase family (*FMO1 *and *KMO*) were significantly differentially expressed. Furthermore, genes involved in conjugation reactions, including the UDP-glucuronosyltransferases (*UGT1A5*, *UGT2A1 *and *UGT2B15*), sulfotransferases (*STE*), N-acetyltransferases (*NAT12*) and glutathione S-transferase were significant, in addition to genes of the 17β-hydroxysteroid dehydrogenase family (*HSD17B2*, *HSD17B4*, *HSD17B11 *and *HSD17B13*), which are known to regulate availability of active steroids. We suggest a novel role for plasma proteins including *AGP *and *ORM1 *in regulating availability of androstenone in pigs. This is the first published microarray experiment describing liver metabolism of androstenone. A number of new candidate genes have been identified, both from phase I and phase II metabolism as well as pathways regulating steroid availability.

## Materials and methods

### Animals

Animals used in this study were Duroc (D) and Norwegian Landrace (NL) boars from NORSVIN's three boar testing stations. The D boars were on average 156 days old at 100 kg live weight compared to the NL boars that were on average 143 days old at 100 kg live weight. The boars were slaughtered on average 14 days later. Tissue samples from liver were frozen in liquid N_2 _immediately after slaughter and stored at -80°C until used for RNA extraction as described below. Samples from adipose tissue were collected from the neck at slaughter and stored at -20°C until used for androstenone measurements. Androstenone levels were measured by a modified time-resolved fluoroimmunoassay at the hormone laboratory, Norwegian School of Veterinary Science (NVH) [61] using an antiserum produced at NVH [62]. The androstenone measurements were performed on more than 2500 boars, and statistical power calculations showed that selecting animals from each tail of the androstenone distribution would yield sufficient power to detect differentially expressed genes with a limited number of arrays. The 30 most extreme boars from each tail of the androstenone level distribution were therefore selected. Due to poor RNA quality for two of the samples, 29 samples were subsequently used from each group. 42 of the animals used were the same individuals as those used in our previous study examining gene expression in boar testis [17], with 16 being new animals as liver samples were not available for all of the previously studied animals. Average androstenone levels for the selected boars were 1.17 ppm and 3.22 ppm for NL and D, respectively. (See additional file 5: Androstenone levels). Average values for the groups were 5.95 ± 2.04 ppm for NL high (NLH), 0.14 ± 0.04 ppm for NL low (NLL), 11.57 ± 3.2 ppm for D high (DH) and 0.37 ± 0.17 ppm for D low (DL). In order to reduce family effects, a maximum of two and three half sibs were chosen from NL and D, respectively. The selected animals were used for expression profiling by microarrays and for the following verification of selected genes by rcPCR.

### Expression profiling using microarrays

The present work utilises and extends methods described in our previous microarray experiment [17]. The porcine cDNA microarrays were produced at the Faculty of Agricultural Sciences, University of Aarhus and contained 27,774 features printed in duplicates. 26,877 features were PCR products amplified from cDNA clones produced by the Sino-Danish Porcine Genome Sequencing project [63,64], and 867 were control features. The 26,877 features represent approximately 20K gene transcripts. Additional information about the porcine cDNA microarray can be found at NCBIs Gene Expression Omnibus (GEO, [65]) using the platform accession number GPL3585. This is a different batch of microarrays compared to the one we used in the testis experiment.

Total RNA was extracted from liver tissue using Qiagen's RNeasy midi kit according to manufacturer's instruction (Qiagen, CA, USA). RNA quantity was measured on a NanoDrop ND-1000 Spectrophotometer (NanoDrop Technologies, DE, USA) and RNA quality was evaluated by the 28S:18S rRNA ratio using a RNA 6000 Nano LabChip^® ^Kit on 2100 Bioanalyzer (Agilent Technologies, CA, USA). The microarray experiment was conducted as a common reference design using RNA purified from liver tissue sampled from an unrelated Danish Landrace × Hampshire pig as a reference. Aminoallyl-cDNA was synthesised from 15 μg of total RNA using the SuperScript indirect cDNA labelling system (Invitrogen Corporation, CA, USA) and labelled using ARES Alexa Fluor labelling kits (Molecular Probes, OR, USA). Amino-modified and fluorescently labelled cDNA was purified using NucleoSpin 96 Extract II PCR Clean-up kits (Macherey-Nagel, Düren, Germany). The individual samples were labelled with Alexa Fluor 647 and the reference was labelled with Alexa Fluor 555. "Green" spike-in RNA from the Lucidea Universal ScoreCard (Amersham Biosciences) was added to the individuals and "red" spike-in RNA was added to the reference. Hybridisation was performed in a Discovery XT hybridisation station (Ventana Discovery Systems, AZ, USA), followed by manual washing and drying by centrifugation. The microarrays were scanned using a ScanArray Express scanner (Perkin Elmer Inc., MA, USA) and image analyses were conducted using GenePix Pro 6 software (version 6.0.1.26, Molecular Devices Corp., CA, USA). Statistical analyses were carried out in R version 2.3.1 [66] using the software package Linear Models for Microarray Analysis (Limma version 2.7.2) [67-69] which is part of the Bioconductor package [70]. Log-transformed ratios of mean foreground intensities (not background corrected) were print tip loess normalised. The duplicate correlation function in Limma was used to consider duplicate printing of each feature. To evaluate the analyses, MA-plots (M = log_2_594/log_2_488, A = (log_2_594 + log_2_488)/2), image plots and box plots were constructed using the Limma tools for visualisation both before and after normalisation (see additional file 6: Boxplots). To assess differential expression, Limma uses linear models in combination with an empirical Bayes method to moderate the standard errors of the estimated log-fold changes [68]. The nominal p-values were corrected for multiple testing by false discovery rates (FDR) using Benjamini and Hochberg approach [71]. Each of the groups DH, DL, NLH and NLL animals were hybridised in individual batches, causing some confounding between androstenone levels and hybridisation batch. However, as neither boxplots nor MA-plots were found to differ between hybridisation batches, it was assumed that the impact of hybridisation batch on the obtained data can be ignored. The top 1% of the genes was considered for further analyses. The array features were mapped to a LocusLink identifier and an annotation package was built using the Bioconductor package AnnBuilder (version 1.9.14). GO terms (p < 0.01 and more than 10 significant genes) were analysed for overrepresentation using the GOHyperG function of the Bioconductor package GOstats (version 1.6.0) [72,73]. More detailed descriptions of the microarray experiments are available at the GEO database through the series accession number GSE 11073.

### Quantitative real competitive PCR analysis

A real competitive PCR (rcPCR) gene expression analysis was used to verify a subset of the results from the microarray study. Quantitative Gene Expression (QGE) was performed using MassARRAY methodology and the iPLEX protocol (Sequenom, CA, USA). Total RNA was isolated from liver using an automatic DNA/RNA extractor (BioRobot M48 workstation; Qiagen; CA, USA). Total RNA was treated with TURBO DNA-free™ (Ambion, Huntingdon, UK) for removal of contaminating DNA and first strand cDNA synthesis was conducted on 0.5 μg total RNA using SuperScript™-II Rnase H^- ^Reverse Transcriptase (Invitrogen, Carlsbad, CA, USA). Assays for the genes included in this study (See additional file 7: Gene transcripts included in the rcPCR analyses) were designed and multiplexed into a single reaction using MassARRAY QGE Assay Design software (Sequenom, CA, USA). The competitor, a synthetic DNA molecule matching the targeted cDNA sequence at all positions except for one single base, served as an internal standard for each transcript. A 10-fold competitor dilution was initially used over a wide range of concentrations to determine an approximate equivalence point. Following this, a 7-fold dilution of competitor from 4.04 × 10^-11 ^to 1.43 × 10^-19 ^was used to achieve accurate quantification. The cDNA and competitor were co-amplified in the same PCR reaction with the following conditions: 95°C for 15 minutes, 45 cycles each of 95°C for 20 second, 56°C for 30 seconds and 72°C for 1 minute, and 72°C for 3 minutes. A clean-up step was performed to remove unincorporated nucleotides. The iPLEX reaction cocktail mix and PCR conditions were according to manufacturer's instructions [74]. Parallel PCR reactions were performed for all samples and the products were printed with two replicates on a SpectroCHIP. The primer extension reaction uses PCR products as templates and generates distinct mass signals for competitor and cDNA-derived products. Mass spectrometric analysis generated signals from which peak areas were calculated. Gene expression levels were analysed using TITAN software version 1.0–13 [75,76] that runs in the R statistical environment. Raw data from the Genotype Analyzer Software (Sequenom) were imported into TITAN and analysed using the default values of linear least squares polynomial regression and 4000 bootstrap replicates. cDNA concentrations were corrected with respect to the housekeeping gene (*TFRC*), and p-values and confidence intervals for fold changes were calculated.

## List of abbreviations

D: Duroc; NL: Norwegian Landrace; DH: Duroc high androstenone; NLH: Norwegian Landrace high androstenone; DL: Duroc low androstenone; NLL: Norwegian Landrace high androstenone; 17β-HSD: 17β-hydroxysteroid dehydrogenase; 3β-HSD: 3β-hydroxysteroid dehydrogenase; AGP: alpha-1-acid glycoprotein; AKR: aldo-keto reductase; AKR1C4: aldo-keto reductase family member 1C4; AKR1D1: aldo-keto reductase family member 1D1; AOX1: aldehyde oxidase; CYB5: cytochrome b5; CYP17: cytochrome P450 family 17; CYP19: cytochrome P450 family 19; CYP27A1: cytochrome P450 family member 27A1; CYP2A: cytochrome P450 family 2A; CYP2A19: cytochrome P450 family member 2A19; CYP2A6: cytochrome P450 family member 2A6; CYP2C19: cytochrome P450 family member 2C19; CYP2C33: cytochrome P450 family member 2C33; CYP2C49: cytochrome P450 family member 2C49; CYP2E1: cytochrome P450 family member 2E1; CYP3A: cytochrome P450 family member 3A; DHRS8: short-chain dehydrogenase/reductase 8; FMO: flavin-containing monooxygenase; FMO1: flavin-containing monooxygenase; GATA4: transcription factor GATA-4; GO: gene ontology; GST: glutathione S-transferase; GSTO1: glutathione S-transferase omega; HSDs: hydroxysteroid dehydrogenases; HSD17B2: hydroxysteroid 17β dehydrogenase 2; HSD17B4: hydroxysteroid 17β dehydrogenase 4; HSD17B11: hydroxysteroid 17β dehydrogenase 11; HSD17B13: hydroxysteroid 17β dehydrogenase 13; HSF1: heat shock transcription factor 1; KMO: kynurenine 3-monooxygenase; MGST1: glutathione S-transferase; NAT: N-acetyltransferase; NAT12: N-acetyltransferase family member 12; NFIX: nuclear factor I/X; ORM1: orosomucoid 1; ORM2: orosomucoid 2; QGE: quantitative gene expression; RORA: RAR-related orphan receptor A; rcPCR: real competitive PCR; SDR: short-chain dehydrogenase/reductase; STE: estrogen sulfotransferase; SULT1A1: sulfotransferase family member 1A1; SULT2E1: sulfotransferase family member 1E1; SULT2A1: sulfotransferase family member 2A1; SULT2B1: sulfotransferase family member 2B1; TCEB3: transcription elongation factor B3; TCF8: transcription factor 8; TDO2: tryptophan 2,3-dioxygenase; TFRC: transferrin receptor; UGT: uridine diphospho-glucuronosyltransferase; UGT1A5: UDP-glucuronosyltransferase family member 1A5; UGT2A1: UDP-glucuronosyltransferase family member 2A1; UGT2B15: UDP-glucuronosyltransferase family member 2B15

## Authors' contributions

MM carried out the microarray experimental work, performed statistical analysis and drafted the paper. SL was involved in planning the project, provided laboratory facilities for rcPCR work and took part in writing the paper. CB was involved in planning the project and was in charge of lab facilities for microarray studies. JH was involved in microarray experimental work and took part in writing the paper. HH carried out bioinformatics work. IB was involved in statistical analysis. TM was involved in power calculations and statistical supervision. EG coordinated the study, was involved in planning the project, carried out rcPCR molecular work and took part in writing the paper.

## Supplementary Material

Additional file 1 Microarray results for Duroc. Gene expression profiling was performed using 58 arrays and the 1% most differentially expressed genes were considered significant. The clone names are sequences with a hit to pig, human, mouse or bovine genes. Some genes are represented by several different clones on the array and may therefore show up more than once in the table, while some have no hits to the abovementioned species. The ID column gives the feature ID on the microarray, M value indicates fold change, t gives the t-statistics, P.value is the nominal p-value and adj.P.value is the FDR corrected p-value. Click here for file

Additional file 2 Microarray results for Norwegian Landrace. Gene expression profiling was performed using 58 arrays and the 1% most differentially expressed genes were considered significant. The clone names are sequences with a hit to pig, human, mouse or bovine genes. Some genes are represented by several different clones on the array and may therefore show up more than once in the table, while some have no hits to the abovementioned species. The ID column gives the feature ID on the microarray, M value indicates fold change, t gives the t-statistics, P.value is the nominal p-value and adj.P.value is the FDR corrected p-value. Click here for file

Additional file 3 Gene ontology (GO) results for the cellular component ontology in Duroc. The top 1% differentially expressed genes in Duroc were analysed for over-represented (p < 0.01) GO terms in the cellular component ontology. Click here for file

Additional file 4 Gene ontology (GO) results for the cellular component ontology in Norwegian Landrace. The top 1% differentially expressed genes in Norwegian Landrace were analysed for over-represented (p < 0.01) GO terms in the cellular component ontology. Click here for file

Additional file 5 Androstenone values. The androstenone values (ppm) in Duroc high (DH), Duroc low (DL), Landrace high (NLH) and Landrace low (LL) animals used in this study. Click here for file

Additional file 6 **Boxplots**. Boxplots of normalised arrays for Duroc (D) and Norwegian Landrace (L). Click here for file

Additional file 7 Gene transcripts included in the rcPCR analyses. Click here for file
