# Supplementary material for: Gene expression profiles in liver of pigs with extreme high and low levels of androstenone
Source: BMC Vet Res. 2008 Aug 6;4:29. doi: 10.1186/1746-6148-4-29 (PMC2535776; doi:10.1186/1746-6148-4-29)
Supplement: Additional file 5 — Androstenone values. The androstenone values (ppm) in Duroc high (DH), Duroc low (DL), Landrace high (NLH) and Landrace low (LL) animals used in this study. [file 1746-6148-4-29-S5.doc]

| DH | DL | LH | LL |
| --- | --- | --- | --- |
| 9.10 | 0.50 | 4.77 | 0.16 |
| 15.33 | 0.19 | 5.71 | 0.19 |
| 12.51 | 0.17 | 4.15 | 0.18 |
| 9.43 | 0.47 | 4.55 | 0.07 |
| 12.51 | 0.48 | 3.88 | 0.15 |
| 12.54 | 0.51 | 7.77 | 0.16 |
| 13.20 | 0.54 | 6.86 | 0.17 |
| 20.52 | 0.22 | 7.02 | 0.17 |
| 9.77 | 0.45 | 5.50 | 0.20 |
| 9.20 | 0.48 | 7.52 | 0.09 |
| 16.92 | 0.39 | 4.26 | 0.15 |
| 8.77 | 0.33 | 3.95 | 0.18 |
| 9.01 | 0.01 | 4.83 | 0.20 |
| 8.97 | 0.10 | 5.83 | 0.17 |
| 9.65 | 0.46 | 8.72 | 0.08 |
| 8.76 | 0.56 | 4.40 | 0.13 |
| 14.40 | 0.31 | 7.88 | 0.08 |
| 8.38 | 0.31 | 6.40 | 0.05 |
| 8.70 | 0.51 | 7.14 | 0.19 |
| 9.92 | 0.50 | 3.77 | 0.17 |
| 8.69 | 0.54 | 6.45 | 0.14 |
| 15.81 | 0.56 | 3.80 | 0.19 |
| 13.47 | 0.39 | 5.09 | 0.07 |
| 15.63 | 0.35 | 5.71 | 0.13 |
| 8.84 | 0.51 | 8.00 | 0.17 |
| 8.68 | 0.01 | 13.40 | 0.15 |
| 12.60 | 0.30 | 5.39 | 0.17 |
| 14.79 | 0.14 | 3.96 | 0.10 |
| 9.52 | 0.52 | 5.94 | 0.13 |
