# Supplementary figures and images for: Gene expression profiles in liver of pigs with extreme high and low levels of androstenone
Source: BMC Vet Res. 2008 Aug 6;4:29. doi: 10.1186/1746-6148-4-29 (PMC2535776; doi:10.1186/1746-6148-4-29)

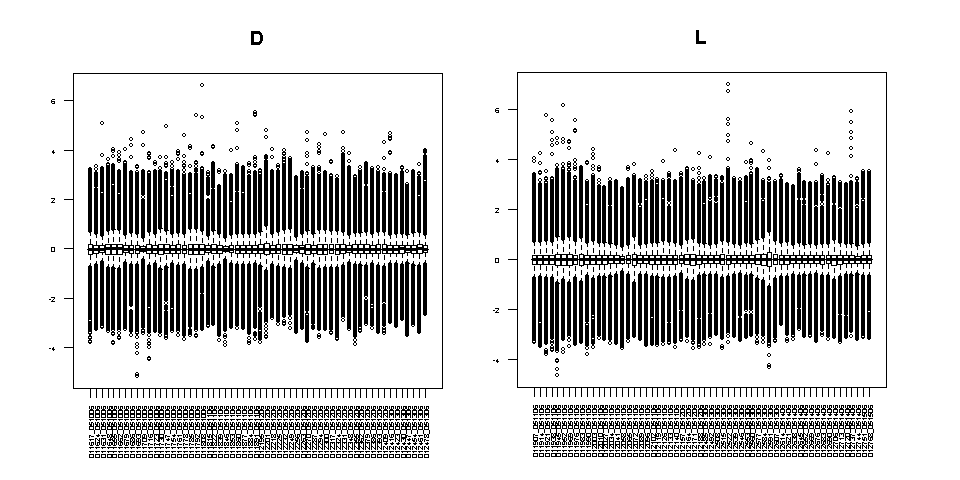

Supplement: Additional file 6 — Boxplots. Boxplots of normalised arrays for Duroc (D) and Norwegian Landrace (L). [file 1746-6148-4-29-S6.png]
