# Supplementary material for: Gene expression profiles in liver of pigs with extreme high and low levels of androstenone
Source: BMC Vet Res. 2008 Aug 6;4:29. doi: 10.1186/1746-6148-4-29 (PMC2535776; doi:10.1186/1746-6148-4-29)
Supplement: Additional file 7 — Gene transcripts included in the rcPCR analyses. [file 1746-6148-4-29-S7.doc]

| Gene | Accession number | Primer sequence |
| --- | --- | --- |
| FMO1 | M32031 | Forward 5’- TGGCAAAAAAGGTGTTCCTC – 3’  Reverse 5’ – TACCCTGAGTCAAAGACACG – 3’  Extension primer 5’ – ccctACACGGCTGATCACCCATGC - 3’ |
| GATA4 | AY115491 | Forward 5’ – AAGAGATGCGTCCCATCAAG – 3’  Reverse 5’ – TGAGAAGGTCTGGGACAGAG – 3’  Extension primer 5’ – caCGGGCTGTCGGCCCACTACGGGCA – 3’ |
| HSD17B13 | DY685093 | Forward 5’ – ATTCCATATTGTTCCAGCAAATT – 3’  Reverse 5’ – TGATACCAGTTTTCCCCACG – 3’  Extension primer5’ – TTGCGGCTGTTGGCTTCC – 3’ |
| HSD17B2 | AK233331 | Forward 5’ – TGCAGAACAGAGGACTGTGGGCT - 3’  Reverse 5’ – ATTGGGTCATGGGAATGAGC – 3’  Extension primer 5’ – TCAACAACGCAGGGATCCTTGGC – 3’ |
| NAT12 | AJ658918 | Forward 5’- ATCTGTCTGAACCCTACTCC – 3’  Reverse 5’ – ACCATGGCCAAGAAGCACAG – 3’  Extension primer 5’ – CACAGCTGTGGCCAG - 3’ |
| TFRC | NM_214001 | Forward 5’ – TGAAGAACCAGATCGCTATG – 3’  Reverse 5’ – TACACTGGACTTTGCAGCTC – 3’  Extension primer 5’ – ACCCCAGGCATCCCTC - 3’ |
